# BIOELECTRICAL VECTOR ANALYSIS IN OBESE ADOLESCENTS

**DOI:** 10.1590/1984-0462/2020/38/2019017

**Published:** 2020-07-13

**Authors:** João Martins de Oliveira, Péricles Soares Bernardes, Guilherme Henrique Costa Serpa, Gabriel Dutra de Jesus Siqueira, Matias Noll, Patrícia Espíndola Mota Venâncio, Viviane Soares

**Affiliations:** a Centro Universitário de Anápolis, Anápolis, GO, Brazil.; b Colégio SESI-Jundiaí, Anápolis, GO, Brazil.; c Universidade Estadual do Norte do Paraná, Jacarezinho, PR, Brazil.; d Universidade Federal de Goiás, Goiânia, GO, Brazil.; e Instituto Federal Goiano, Ceres, GO, Brazil.

**Keywords:** Electric impedance, Hydration, Obesity, Adolescents, Impedância elétrica, Hidratação, Obesidade, Adolescentes

## Abstract

**Objective:**

To evaluate the hydration of obese and non-obese adolescents by vectorial bioimpedance analysis, in addition to verifying the associations between obesity and bioelectrical impedance vectorial analysis (BIVA) parameters.

**Methods:**

A cross-sectional study with adolescents between 14 and 18 years old (n=489, 300 boys and 189 girls). Electric bioimpedance (BIA; Quantum_II, RJL system, Rome, Italy) provided resistance and reactance parameters to calculate phase angle (PA), fat-free mass (FFM) and body fat (%BF). The confidence ellipses were compared, and the construction of the tolerance ellipses allowed individual and qualitative evaluation of the vectors and classification in dehydrated, normohydrated and hyperhydrated.

**Results:**

78 obese and 411 eutrophic adolescents participated. Resistance (p<0.001) and reactance (p<0.001) and their normalization by stature (p<0.001) were reduced in the obese, whereas the PA was higher (p=0.003). %BF was 11.3% higher in obese adolescents. The main vector of the obese, both male (D=1.38; p<0.001) and female (D=1.49; p<0.001), indicated greater hydration. The ellipse of tolerance of the total sample showed that 25 (32.1%) were hyperhydrated and 02 (2.6%) vectors positioned in the sense of dehydration. A total of 17 (53.2%) girls and 16 (34.8%) boys were hyperhydrated. Logistic regression showed an inverse relation of BMI with resistance (p<0.001), reactance (p<0.001) and both normalized by stature. Adolescents with increased PA (p<0.001) were twice as likely to present obesity.

**Conclusions:**

Obese adolescents were hyperhydrated and there was an inverse relationship of BMI with resistance and direct with PA.

## RESUMO

**Objetivo:**

Avaliar a hidratação de adolescentes obesos e não obesos por meio dos gráficos da análise vetorial por bioimpedância (BIVA), além de verificar as associações entre a obesidade e os parâmetros da BIVA.

**Métodos:**

Estudo transversal realizado com adolescentes entre 14 e 18 anos (n=489, 300 meninos e 189 meninas). A bioimpedância elétrica (BIA; Quantum_II, RJL system, Roma, Itália) ofereceu os parâmetros de resistência e reactância para calcular o ângulo de fase (AF), a massa livre de gordura (MLG) e a gordura corporal (GC). As elipses de confiança foram comparadas e a construção das elipses de tolerância permitiu a avaliação individual e a qualitativa dos vetores e, ainda, a classificação em desidratados, normohidratados e hiper-hidratados.

**Resultados:**

Participaram do estudo 78 adolescentes obesos e 411 eutróficos. A resistência (p<0,001), a reactância (p<0,001) e a normalização desses pela estatura (p<0,001) estavam reduzidas nos obesos, enquanto o AF foi superior (p=0,003). O percentual de gordura corporal (%GC) foi 11,3% maior nos adolescentes obesos. O vetor principal dos obesos, tanto masculino (Distância de Mahalanobis (D)=1,38; p<0,001) quanto feminino (D=1,49; p<0,001), indicou maior hidratação. A elipse de tolerância da amostra total mostrou que 25 adolescentes (32,1%) estavam hiper-hidratados e dois (2,6%) vetores posicionados no sentido de desidratação. Dezessete (53,2%) meninas e 16 (34,8%) meninos estavam hiper-hidratados. A regressão logística mostrou relação inversa do índice de massa corpórea (IMC) com a resistência (p<0,001) e a reactância (p<0,001), ambas normalizadas pela estatura. Os adolescentes com aumento do AF (p<0,001) apresentaram duas vezes mais chances de serem classificados como obesos.

**Conclusões:**

Os adolescentes obesos estavam hiper-hidratados e houve relação inversa do IMC com a resistência e direta com o AF.

## INTRODUCTION

Obesity is considered a worldwide public health problem, being one of the main factors for the acquisition of non-communicable diseases (NCDs).^1^ In a study carried out in 195 countries, a prevalence of 5% (107.7 million) of overweight/obesity was found in children/adolescents.^2^

Obesity does not only affect the adult population. The multicenter Study of Cardiovascular Risks in Adolescents (ERICA) showed that 17.1% of Brazilian adolescents are overweight and 8.4% are considered obese, which is a worrying factor for quality of life and survival in the Brazilian population.^3^

Anthropometric measurements are widely used to identify overweight/obese adolescents due to their low cost and the possibility of large-scale evaluation. The body mass index (BMI) is considered a general health marker and is most frequently used to classify adolescents as eutrophic, overweight/obese.^4^ In addition to anthropometric measurements, bioimpedance analysis (BIA) is widely used in the clinical outcome for being a practical, non-invasive, fast and relatively low-cost method.^5^ ^,^ ^6^ ^,^ ^7^ BIA is able to estimate both body fat (BF) and muscle mass and the amount of fluids in the body, differentiating intracellular from extracellular water using regression equations and the resistance (R), reactance (Xc) and phase angle (PA) parameters.^4^ ^,^ ^5^ There are numerous validated equations in the literature, but differences in clinical conditions, ethnicity of groups and age can underestimate and overestimate body composition and interpretation of results, which can compromise the assessment of hydration.^8^ On the other hand, bioelectrical impedance vector analysis (BIVA) is a tool created to complement and minimize BIA problems, not requiring regression equations to measure body composition. The evaluation for hydration and cell mass is carried out in a qualitative way and consists of the construction of a graph whose values of R and Xc are normalized by height (H) (R/H and Xc/H, respectively) and plotted, originating a graph R/Xc.^9^ The interpretation is based on the length of the impedance vectors, their ellipses and the PA. The longest and most resistant vector characterizes dehydration, whereas the short vector indicates hyperhydration (less resistance), and if greater PA occurs, it is related to a better nutritional status.^10^ ^,^ ^11^

BIVA has been seen as an important tool to check hydration status and cell mass, and this is why it has been used as a prognosis in various clinical conditions, such as chronic renal failure, patients undergoing hemodialysis, heart failure, some types of cancer, anorexia nervosa, among others.^12^ ^,^ ^13^ ^,^ ^14^ ^,^ ^15^ Only one study was carried out with women undergoing weight loss using BIVA,^16^ but the application of this evaluation method in obese adolescents is the first described in the literature. Based on such information, the objective of the present study was to evaluate the hydration of obese and non-obese adolescents using BIVA graphs, in addition to verifying the associations between obesity and BIVA parameters.

## METHOD

This is an observational cross-sectional study. Research was carried out in a high school with approximately 563 students enrolled in the morning shift, between August and November 2017, and aged between 14 and 18 years old. Participants in the study were students who did not eat in the three hours prior to the exam and did not perform any physical activity the day before. Students with pathologies or clinical conditions (acute asthma, heart disease and high blood pressure) that prevented evaluations from being performed and students with cognitive impairment were excluded from the study. The sample calculation was made with the GPower^®^ software (version 3.1), considering a sampling power of 80%, an average effect size of 0.15 and a significance level of 5%. For that, 78 obese adolescents would be needed. Recruitment was carried out for convenience and all eligible students were evaluated until reaching the required obese sample. Thus, the total sample was 489 students (300 boys [61.3%] and 189 girls [38.7%]), with 78 obese adolescents and 411 eutrophic ones.

The adolescents and their parents/guardians signed the informed consent form, and the project was approved by the Research Ethics Committee (CEP) of Centro Universitário-UniEvangélica, No. 2.064.213/2017.

An identification form was filled out by the adolescents in the classroom with information on age, sex and sexual maturity, according to Tanner’s criteria.^17^ As to sexual maturity, the adolescents were asked to mark in a specific place on the identification of the presence of pubic hair, the development of genitals, menarche in girls and ejaculation in boys.

In order to measure body mass, a Filizola digital scale (model 2096 PP, São Paulo, Brazil) was used, with a resolution scale of 0.1 kg and a capacity of up to 150 kg. H was measured in meters (m) using a stadiometer (Sanny, São Paulo, Brazil). BMI was calculated by dividing body mass by H squared. The cutoff points for classifying adolescents as eutrophic (Z score between -2 and +1) and obese (Z score> +1) followed the guidelines of the World Health Organization (WHO).^4^ In the sample, overweight/obese adolescents were assessed.

BIA was performed with a tetrapolar device (Quantum_II, RJL system, Rome, Italy) that uses an excitation current (500 to 800 µA) at 50 Hz. The electrodes were positioned in the dorsal region of the hand (one between the head of the ulna and the radius, and the other in the proximal phalanx of the third finger) and in the foot (an electrode between the medial and lateral malleoli and another in the third metatarsal region) after cleaning the region with 70% alcohol. The measurements of R and Xc were performed in duplicate and the highest value was used for analysis. The PA was calculated using the Xc/R * 180º/π.^18^ arctangent. These variables represent the hydration status and the soft tissue cell mass and are related to the integrity, permeability and intra and extracellular spaces.^18^ ^,^ ^19^

The percentage of body fat (%BF) and the FFM were calculated according to the BIA’s parameters with the equations described below:^20^

- FFM
  - $$Boys:\;FFM\;=\;-10.678\;+\;0.262\;(body\;mass\;in\;kg)\;+\;0.45*({height}^{2}/R)$$
  - $$Girls:\;FFM\;=\;-9.529\;+\;0.168\;(body\;mass\;in\;kg)\;+\;0.696*({height}^{2}/R)$$
- %BF
  - BF = body mass - FFM
  - %BF = BF/body mass

The vector analysis by BIA (BIVA) was performed using the BIVA software, developed by Piccoli et al.,^9^ and uses the plotting of R by Xc, normalized by H, in order to build the confidence ellipses. This analysis allows for a qualitative assessment, and normalization by H indicates the length of the vector, thus not depending on body size.^9^ The confidence ellipses are compared and the interpretation occurs as follows: the length of the vector on the major axis indicates hydration status - if there is an increase in resistance, the condition of dehydration is considered, so the vector will be long, whereas the reduction in resistance establishes hyperhydration, the vector being short.^19^ The distance (D) of Mahalanobis is what confers similarity between confidence ellipses.

The tolerance ellipses establish the intervals of 50, 75 and 95%, which are used to analyze the individual vectors from a reference population. In the present study, eutrophic adolescents were the reference for the analysis of individual vectors. In BIVA, the long vector provides information on the hydration status, whereas the smaller vector indicates a difference in cell mass in soft tissues. The left side of the tolerance ellipse suggests more cell mass, and the upper and lower quadrants have been validated for athletic and obese populations, respectively.^9^ The right side of the ellipse characterizes individuals with less cell mass, and the quadrants are validated for individuals as thin and cachectic (upper and lower, respectively).^9^ The 75% tolerance ellipse is considered the reference to indicate normohydration in the vector analysis; above, it is considered dehydrated; and, below, hyperhydrated.^21^

The data were described with mean, standard deviation and graphs. To verify the normality of the data, the Kolmogorov-Smirnov or Shapiro-Wilk test was used when needed. In order to compare the variables between obese and eutrophic adolescents, Student’s t test was used for independent samples. For the vector analysis, Hotelling’s T-squared test was performed, the univariate analysis (F test) and the D between the confidence ellipses was determined by the Mahalanobis test. Logistic regression, using the Stepwise method, assessed the relation between BMI (dependent variable) and BIA and BIVA parameters (independent variables), adjusted for gender, age and sexual maturity (present or absent). The significance level considered was <0.05. The data were analyzed using the Statistical Package for the Social Sciences (SPSS) software, version 21.0 (Chicago, United States), and BIVA 2002 (University of Padova, Padova, Italy).

## RESULTS

The prevalence of obesity in the population was 16%, with a predominance of girls (17%) when compared to boys (15%). The characteristics of participants are described in Table 1. Sexual maturity was present in 219 (73%) boys and 163 (86.2%) girls.

**Table 1:** Basic characteristics of adolescents (n=489).

	Obese (n=78)	Eutrophic (n=411)	∆	*d*	p-value*
Gender (male/female)	46/32	254/157			
Age (years old)	16.2±0.9	16.0±1.0	0.2	0.21	0.16
Body mass (kg)	81.6±12.4	56.0±8.6	25.6	2.40	<0.001
Height (cm)	168.8±8.6	167.1±8.7	1.7	0.20	0.11
BMI (kg/m^2^)	28.6±3.4	20.1±2.2	8.5	2.97	<0.001

∆: variation among groups; *d*: Cohen’s effect size; BMI: body mass index; *p<0.05.

R (p <0.001), R/H (p<0.001), Xc (p<0.001) and Xc/H (p<0.001) were reduced in obese adolescents, whereas PA was higher (p=0.003) (Table 2). Obese adolescents had a %BF 11.3% higher than non-obese adolescents. In relation to the percentage of FFM, obese adolescents had 11.3% less when compared to eutrophic adolescents (p<0.001).

**Figure 1:**
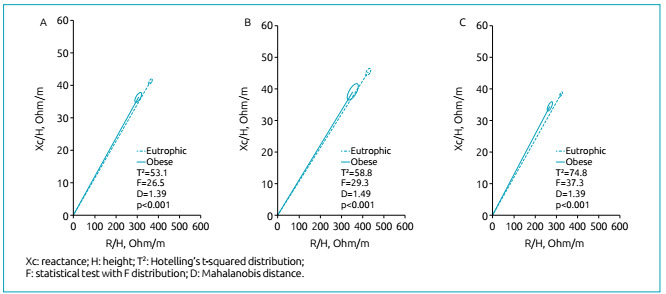
Comparison of confidence ellipses between obese and eutrophic individuals according to gender: (A) total sample; (B) female adolescents; and (C) male adolescents.

The confidence ellipse of the total sample indicated that obese adolescents were more hydrated (D=0.9; p<0.001) (Figure 1A). Likewise, when the comparison was made according to gender, the main vector of obese adolescents, both boys (D=1.38; p<0.001) and girls (D=1.49; p<0.001), was directed towards hydration (Figures 1B and 1C). The Mahalanobis distance parameter indicates how the main vectors are distant from each other, demonstrating that the more distant, the greater the difference.

**Table 2:** Adolescents’ bioelectrical impedance (n=489).

Bioelectrical impedance	Obese (n=78)	Eutrophic (n=411)	∆	*d*	p-value*
Resistance (ohms)	512.8±78.6	602.1±91.3	-89.3	1.05	<0.001
Resistance/height (ohms)	305.7±56.5	362.7±66.4	-57.0	0.92	<0.001
Reactance (ohms)	61.1±7.3	68.3±8.3	-7.2	0.92	<0.001
Reactance/height (ohms)	36.3±5.1	41.1±6.2	-4.7	0.85	<0.001
Phase angle (º)	6.9±0.9	6.5±0.8	0.3	0.47	0.003
Body fat (kg)	27.2±7.9	12.4±4.6	14.8	2.29	<0.001
Body fat (%)	33.4±7.9	22.1±7.4	11.3	1.48	<0.001
Fat-free mass (kg)	54.3±10.2	43.6±7.9	10.7	1.17	<0.001
Fat-free mass (%)	66.6±7.9	77.9±7.4	-11.3	1.48	<0.001

∆: variation among groups; *d*: Cohen’s effect size; *data for p<0.05.

**Figure 2:**
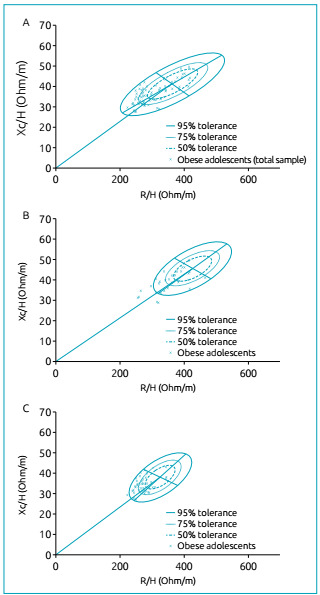
Distribution of individual vectors of obese adolescents according to gender: (A) total sample; (B) female; and (C) male.

The tolerance ellipses (Figure 2A) revealed that of the 78 adolescents classified as obese, 17 (21.8%) were hyperhydrated and located in the obesity quadrant (lower left quadrant); eight (10.3%), hyperhydrated in the lower right quadrant; and two (2.56%), in the vector positioned in the direction of dehydration (upper right quadrant). It is worth mentioning that the vectors positioned within the 75% ellipse were considered the norm for normohydration.

The reference ellipse for the assessment of obese adolescents according to gender was constructed with the sample of girls and boys separately. Among female adolescents, ten (31.3%) were hyperhydrated and located in the obesity quadrant (lower left), outside the 75% ellipse, and seven (21.9%) in the cachexia quadrant (lower left) (Figure 2B). Obese male adolescents had their vectors positioned within the quadrant validated for obesity, but 13 (28.3%) adolescents were hyperhydrated and three (6.5%), in the lower right quadrant (Figure 2C).

The logistic regression models showed a relation between BMI (dependent variable) and BIA parameters (Table 3). R (p<0.001), Xc (p<0.001) and both normalized by H showed an inverse relation with BMI, whereas adolescents with increased PA (p<0.001) were twice as likely to have obesity. All models were adjusted for gender, sexual maturity and age.

**Table 3:** Logistic regression of the relation between body mass index (dependent variable) and bioelectrical impedance parameters (independent variables).

	BMI		
	OR	95%CI	p-value
Resistance (R)	0.97	0.97-0.98	<0.001
Reactance (Xc)	0.86	0.83-0.90	<0.001
Resistance/height (R/H)	0.96	0.95-0.97	<0.001
Reactance/height (Xc/H)	0.80	0.75-0.85	<0.001
Phase angle (PA)	2.02	1.4-2.91	<0.001

BMI: body mass index; OR: *Odds Ratio*; 95%CI: 95% confidence interval. Adjusted for age, gender and sexual maturity. Data for p<0.05.

## DISCUSSION

The present study demonstrated that BIA markers - R, Xc, R/H and Xc/h - were lower for obese adolescents, whereas PA was higher. According to BIVA, the confidence ellipses demonstrated that, both in the total sample and in the sample stratified by gender, the main vector of obese adolescents indicated greater hydration, when compared to eutrophic ones. In the tolerance ellipses, which assess individually and qualitatively, most were positioned in the obesity quadrant, within the 75% limit. However, among girls, there were adolescents who were positioned between the limits of 75 and 95%, in the sense of hyperhydration. The logistic models adjusted for age, gender and sexual maturation showed an inverse relation between BMI and R, Xc, R/H and Xc/H, and a direct relation with PA.

Obese adolescents had a higher percentage of fat, as expected,^22^ and the parameters of BIA and BIVA - R, R/H, Xc and Xc/H - were lower, when compared to eutrophic adolescents. These results were similar to those from studies carried out with Brazilian healthy and athletic adolescents.^23^ ^,^ ^24^ In adolescent who where athletes, the parameters of BIA and BIVA were compared according to bone maturity (“in time”, “early” and “delayed”) and were similar to those of the present study.^24^ The results suggest less resistance to the passage of electrical current in the tissue, thus enabling greater intra and extracellular ion transit through the membrane.^7^ ^,^ ^21^ A smaller Xc in obese adolescents indicates that the delay in the conductivity of the current was lower between the cell membrane and the tissue, when compared to eutrophic adolescents,^21^ which reveals a greater number of conductors. In the sample studied, the PA of obese adolescents was higher, and this parameter indicates better health and integrity of the cell membrane, in addition to greater body cell mass,^19^ when considering the hydration aspect.

The analysis of the BIVA confidence ellipses among obese and eutrophic adolescents, according to gender, showed that those obese were more hydrated. In the literature, this increase in hydration of obese individuals, when compared to healthy individuals, was demonstrated in a study that followed the loss of body mass of adult women.^16^ The shorter vector of obese adolescents represents a reduction in R and a greater net overload,^19^ and D between the mean vectors (Mahalanobis distance) confirmed the significant difference between the confidence ellipses. Probably, the greater hydration of obese adolescents is due to the expansion of extracellular water (EW).^25^ A study carried out with the measurement of total body water (TBW) (deuterium oxide solution) and EW (sodium bromide dilution) showed that obese children and adolescents have a higher TBW. However, when comparing the hydration of those obese, in percentage, with the body mass, eutrophic children were more hydrated. EW was greater in obese children, whereas intracellular water did not change, suggesting that obesity is associated to the expansion of EW and, thus, that homeostasis disorders start early.^25^

The tolerance ellipses demonstrated that most of the obese adolescents, of both genders, had their vectors positioned in the direction of hyperhydration (Figure 2A - lower left quadrant), beyond the 75% limit. This condition of greater hydration was also seen in young elite swimmers when compared to a reference population. These athletes, evaluated pre- and post-training, had their vectors plotted outside the 75% limit.^26^ Recently, De Mateo-Silleras et al.^27^ showed that children between five and 18 with overweight/obesity also presented their individual vectors in the lower left quadrant, demonstrating to be a promising method in pediatrics, since electrical data standardized by H are used, without the need for predictive models. BIVA, bioelectrical impedance vector analysis, is a method that differs from others in its ability to analyze and monitor the body composition of individuals and compare groups of different clinical conditions,^13^ ^,^ ^28^ not depending on body geometry and regression equations.^21^

Obese adolescents of both genders had their vectors in the direction of hyperhydration positioned in the lower left quadrant, validated for obese individuals according to the classification ellipse of Piccoli et al.^9^ This qualitative analysis showed the lateral migration of the vectors, indicating high Xc and, consequently, the presence of greater dielectric tissue mass.^19^ It is noteworthy that the control of body mass is needed for these obese adolescents, given that the net overload when associated to aging may lead to heart failure.^29^

There was an inverse relation between R/H and BMI. However, we must remember BMI is not a parameter of body composition, but has a high relationship with several markers that predict muscle and fat tissue.^29^ This parameter is considered a general health marker and is even used in the multivariate analysis of BIVA (Hotelling’s t-squared distribution). The direct relation between PA and BMI has already been demonstrated in the literature, but with lower values than those from the present study.^24^ ^,^ ^30^ On the other hand, there is evidence in the literature of an inverse relation between PA and BMI of obese individuals when the BMI was higher than 30 kg/m^2^.^31^ It is important to note that PA has a direct relation with muscle mass, and that the older the age, consequently, the lower the PA.^19^ PA has been pointed out as a parameter that indicates cellular health, in which high values indicate greater cellularity, cell function and membrane integrity. Its importance is being suggested by some authors, showing it to be an important tool to assess the patient’s clinical status, indicated as a prognostic factor of some diseases.^32^ ^,^ ^33^

In the adolescents from the present study, this increase (greater PA) indicates, proportionally, greater Xc for a given R. This condition is attributed to greater hydration due to water overload or the increase in the ratio between EW and intracellular water from adipose tissue.^34^ This would occur because of the increase in EW. Clinically, high PA values would be a protective factor for adolescents, but with increasing age and the low level of chronic obesity inflammation, this protective relation could not be maintained. In addition, PA can be used as a marker of nutritional status, considering it relates to protein dosages, such as creatinine, total proteins and albumin.^19^

The study has strong points. The number of adolescents evaluated was greater than that expected in the sample calculation (78 obese and 411 non-obese), with the participation of adolescents of all age groups. To our knowledge, this is the first study that evaluated the changes in the hydration status of obese adolescents of both genders with BIVA. A limitation related to the present study was the recruitment of participants for convenience. It was not possible to verify the cause-effect relationship due to the study design carried out, and new papers are expected to be able to generalize the results. However, the data found in the present study can be used as a starting point for further research, including longitudinal studies, in order to monitor chronic clinical conditions.

According to the results found, obese adolescents had low R, Xc and FFM, whereas %BF and PA were higher. Confidence ellipses showed that obese individuals were hyperhydrated when compared to eutrophic individuals. Most individual vectors were positioned in the lower quadrant (right and left), which is validated in the literature for obesity. There was an inverse relation between BMI and R, and a direct relation with PA, suggesting that obese adolescents are more hydrated than eutrophic ones.
